# Genome-wide association study in hexaploid wheat identifies novel genomic regions associated with resistance to root lesion nematode (*Pratylenchus thornei*)

**DOI:** 10.1038/s41598-021-80996-0

**Published:** 2021-02-11

**Authors:** Deepak Kumar, Shiveta Sharma, Rajiv Sharma, Saksham Pundir, Vikas Kumar Singh, Deepti Chaturvedi, Bansa Singh, Sundeep Kumar, Shailendra Sharma

**Affiliations:** 1 Department of Genetics and Plant Breeding, Chaudhary Charan Singh University (CCSU), Meerut, Uttar Pradesh 250 004 India; 2 Department of Botany, Chaudhary Charan Singh University (CCSU), Meerut, Uttar Pradesh 250 004 India; 3 Scotland’s Rural College (SRUC), Peter Wilson Building, West Mains Road, Edinburgh, EH9 3JG UK; 4 Division of Crop Protection, Indian Institute of Pulses Research (IIPR), Kanpur, Uttar Pradesh 208 024 India; 5 Division of Genomic Resources, National Bureau of Plant Genetic Resources (NBPGR), Pusa campus, New Delhi, 110 012 India

**Keywords:** Genetics, Plant sciences

## Abstract

Root lesion nematode (RLN; *Pratylenchus thornei*) causes extensive yield losses in wheat worldwide and thus pose serious threat to global food security. Reliance on fumigants (such as methyl bromide) and nematicides for crop protection has been discouraged due to environmental concerns. Hence, alternative environment friendly control measures like finding and deployment of resistance genes against *Pratylenchus thornei* are of significant importance. In the present study, genome-wide association study (GWAS) was performed using single-locus and multi-locus methods. In total, 143 wheat genotypes collected from pan-Indian wheat cultivation states were used for nematode screening. Genotypic data consisted of  > 7K SNPs with known genetic positions on the high-density consensus map was used for association analysis. Principal component analysis indicated the existence of sub-populations with no major structuring of populations due to the origin. Altogether, 25 significant marker trait associations were detected with − log10 (*p* value) > 4.0. Three large linkage disequilibrium blocks and the corresponding haplotypes were found to be associated with significant SNPs. In total, 37 candidate genes with nine genes having a putative role in disease resistance (F-box-like domain superfamily, Leucine-rich repeat, cysteine-containing subtype, Cytochrome P450 superfamily, Zinc finger C2H2-type, RING/FYVE/PHD-type, etc.) were identified. Genomic selection was conducted to investigate how well one could predict the phenotype of the nematode count without performing the screening experiments. Prediction value of r = 0.40 to 0.44 was observed when 56 to 70% of the population was used as a training set. This is the first report where GWAS has been conducted to find resistance against root lesion nematode (*P. thornei*) in Indian wheat germplasm.

## Introduction

Bread wheat (*Triticum aestivum* L.) is one of the most important cereal crops cultivated globally and is a major source of calories for the growing world population^1^. Green revolutions of the 1960s and 1980s led to the significant improvement in wheat production particularly in South-East Asia^1,2^. Record global wheat production of 761.5 million tons was achieved in 2019 which is expected to further increase by 2020^3^. However, the current trends in wheat production increase appear to be insufficient for feeding the population of nine billion people predicted for 2050^4^. To meet the growing human needs without increasing the area of cultivated land, which is simply not available, wheat grain production must increase. Although production is increasing, yields are still vulnerable to a variety of biotic and abiotic factors. Among biotic factors, plant-parasitic nematodes (PPNs) are one such limiting factor that cause losses of ~ 12.6% among different crop plants, representing an annual monetary loss of 216 billion US$^5^. The most common damaging PPNs are the root-knot nematodes (RKNs), cereal cyst nematodes (CCNs) and root lesion nematodes (RLNs). RLNs of genus *Pratylenchus* are obligatory endoparasites. *Pratylenchus* spp. are polyphagous in nature and feed on a wide range of crops including cereals, vegetables, legumes, coffee, etc. These are migratory endoparasites which penetrate into the root cortex, using their stylet and release cell wall degrading enzymes and migrate inter-cellularly^6^. Due to damage in the root cells, plants are unable to uptake water and nutrients from soil properly, producing symptoms similar to nutrient and water deficiency^7,8^. Completion of their life cycle takes 3–8 weeks depending on the species and conditions^7^. After embryogenesis and development, the first stage juvenile (J1) molts to the second stage juvenile (J2) within the egg which further hatches from the egg^9^. All juveniles and adult stages are mobile and can enter and leave the roots. They are distributed worldwide and have been reported on wheat cultivation in Syria, Mexico, Canada, Israel, Yugoslavia, Morocco, Iran, India, Turkey, Pakistan, Algeria, Italy, Australia, and USA^10^. Several of these countries recorded significant wheat yield loss due to the *P. thornei* infection^11^. In terms of economic importance, yield loss up to 62% has been recorded, due to *P. thornei*, in countries like Australia^12^.

*P. thornei* is an important and well-known pest of chickpea in India and its high population has been reported in many states including Uttar Pradesh^13–16^. However, *P. thornei* has also been reported to damage rice–wheat–legume cropping sequence and hence may be considered as an emerging pathogen of wheat and rice in India^17,18^. No significant genetic studies have been done in India on wheat and *P. thornei* interaction, except for a few survey reports^19,20^. As far as wheat yield loss is concerned, up to the best of our knowledge, any major study to estimate the wheat yield loss in India caused by *P. thornei* has not been performed yet.

Application of nematicides is quite effective in controlling nematodes^21,22^. However, nematicides such as methyl bromide and aldicarb is not in practice for agricultural use due to human health and environmental concerns^23,24^. Similar to other PPNs, *P. thornei* can also be managed through an integrated approach including farm hygiene, growing of resistant or tolerant varieties of wheat and crop rotation with non-host crops^25,26^. Growing resistant or tolerant cultivars is a more effective, economic and eco-friendly method to control the nematodes. There are several reports indicating that nematode’s reproduction and densities can be restricted by deploying resistant cultivars, while a wheat cultivar tolerant to *P. thornei* maintains the growth and yield, even after maintaining nematode reproduction^27–30^. Even though, complete resistance to *P. thornei* is currently not available for wheat^31^. The bread wheat line GS50a is the first known source of partial resistance to *P. thornei* obtained from a field of severely affected wheat of the variety Gatcher^32^. Since this line shows partial resistance to *P. thornei,* other germplasm screenings were also conducted (Middle Eastern landraces, wild wheat progenitors, synthetic wheat and CIMMYT wheat accessions) to identify another source of resistance against *P. thornei*^31,33–35^. Several quantitative trait loci (QTLs) linked to *P. thornei* resistance have also been identified in wheat, and these resistance sources could be incorporated into wheat breeding programs^36,37^. Three major QTLs for *P. thornei* resistance on chromosome 2BS, 6DS and 6DL were identified in the synthetic hexaploid wheat CPI133872^37^. Toktay et al.^38^ reported QTLs for *P. thornei* resistance on chromosome 1B, 2B, 3B, 4D and 6D. Eight QTLs for *P. thornei* resistance were mapped on wheat chromosome 2A, 2B, 2D, 5D and 6D^36^. Out of these, two major QTLs namely “*QRlnt.sk-2B*” on chromosome 2B and “*QRlnt.sk-6D*” on chromosome 6D were fine mapped^39^. Only the single gene *Rlnn1* originating from Australian spring wheat cv. Excalibur for *P. neglectus* resistance was mapped on the chromosome 7AL in a doubled haploid population^40^.

Association mapping (AM) or linkage disequilibrium (LD) mapping is used to search genotypic-phenotypic correlations in unrelated individuals. It could be a promising strategy for the identification of significant marker-trait associations for nematodes resistance. GWAS uses all recombination events that have occurred historically within the crop, resulting in much higher mapping resolution by exploiting the historical linkage disequilibrium^41^. However, not many GWAS studies have been conducted in wheat to find resistance against *P. thornei*. Dababat et al.^42^ found nine significant MTAs using GWAS associated with *P. thornei* resistance on chromosomes 1D, 2A, 3B, 5B and 7A.

The present study was conducted to find out the novel sources of resistance i.e., MTAs and candidate genes significantly associated with *P. thornei* resistance in wheat genotypes which have been predominantly collected from India. Mainly single-locus and multi-locus GWAS methods were compared in order to identify significant marker-trait associations (MTAs). We also explored the overlap of the identified MTAs and the known QTLs from previous studies to locate the novel genomic regions which could be targeted in plant breeding. Lastly, we have conducted the genomic-selection on the genotypes to investigate the efficiency of genomic-selection in predicting the complex trait such as RLN resistance. This is the first GWAS study from India to find resistance against *P. thornei* in wheat.

## Results

### Phenotypic analysis

To identify the region of wheat genome contributing resistance to *P. thornei*, 143 wheat genotypes predominantly from India were screened for nematode resistance. Screening experiments were carried out over two years by growing wheat genotypes in five different batches with total 5–7 replications. A histogram depicting the distribution of nematodes among genotypes is shown in Supplementary Fig. S1. The restricted maximum likelihood (REML) analysis of the nematode count revealed significant effect of the genotypes which was ascertained by comparing the models wherein batch and replications were considered as random terms. Further, the batch effect was higher than the replication effects which are given in Supplementary Table S1. Thus, the best linear unbiased predictions (BLUPs) were calculated using batch and genotypes as a random term. We obtained a high variation for the nematode counts across the screened genotypes with minimum = 33, maximum = 13,503 and the coefficient of variation (CV) of 62.96%. The heritability value of the nematode counts was moderate (h^2^ = 0.55) (Supplementary Table S2).

### SNP markers statistics

**Figure 1:**
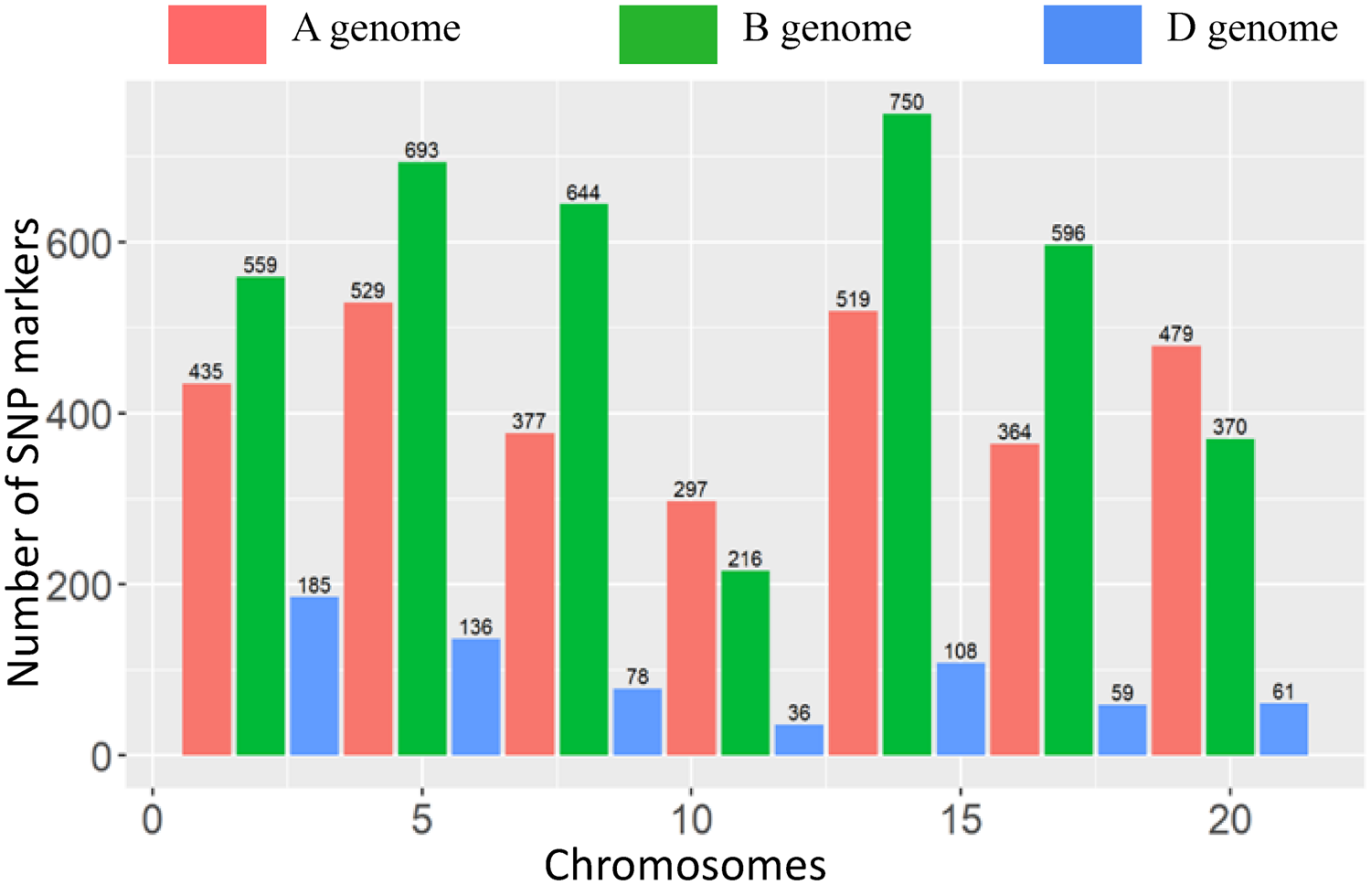
Distribution of SNP markers across all seven homoeologous chromosomes of wheat.

In the present study, a total of 16,406 SNP markers were extracted after initial filtering for the quality on the chip and the missing data. This number was further reduced to 7,491 SNP markers, after filtering SNP markers with a missing rate higher than 10%, minor allele frequency (MAF) lower than 5% and heterozygosity higher than 10%. Marker coverage of the SNPs over the chromosomes is shown in Fig. 1. Marker density was found highest for the B genome followed by A and D genome (Supplementary Fig. S2). The maximum number of markers were found on chromosome 5B while chromosome 4D spanned the minimum number of markers.

### Principal component analysis

**Figure 2:**
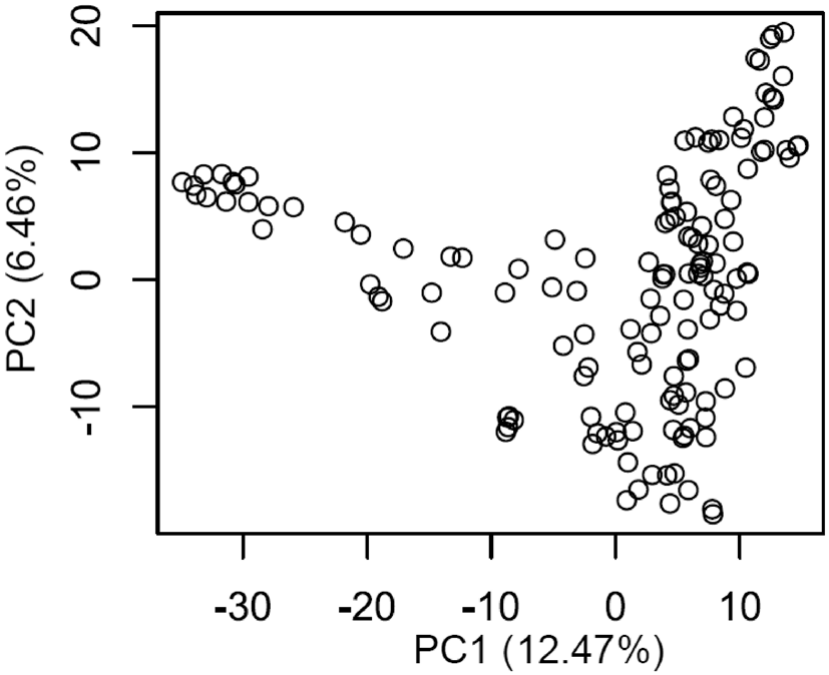
Principle component analysis (PCA), showing the distribution of genotypes along with two components, obtained using 7,491 polymorphic SNP markers and 143 wheat genotypes.

To estimate population structure, a PCA was conducted with 7,491 polymorphic SNP markers and 143 bread wheat genotypes (Fig. 2). PC1 explained 12.47% variation and PC2 explained 6.46% variation. Six genotypes clustered very close to each other although none of them shared any similarity in terms of the origin of the germplasm (these genotypes were originated from Maharashtra, Jharkhand, New-Delhi and Madhya Pradesh states of India). We also explored further whether the structure of the population is associated with the nematode resistance. Genotypes clustered towards the left side were associated with low nematode counts whereas the right side genotypes were susceptible with higher nematode count (r = 0.11, *p* value  < 0.05). Further, a significant correlation was obtained for phenotypes with PC4-axis (− 0.15, *p* value  < 0.05) wherein genotypes clustered towards the lower axis were resistant compared to the upper axis (data not shown).

### Linkage disequilibrium (LD)

**Figure 3:**
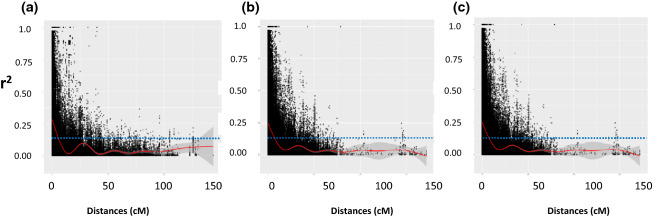
Linkage disequilibrium decay measured as r-squared against the genetic distance between pairs of SNPs. LOESS curve was fitted showing that LD decays with genetic map distance and dashed line indicates the derived threshold for LD due to linkage. (**a**) LD decay in A genome (**b**) LD decay in B genome (**c**) LD decay in D genome.

Faster LD-decay was observed for the A-genome (5–7 cM) compared to the B and D-genome (8–9 cM) of the panel. However, local spikes in the long-range LD regions were also observed that indicates the population structure of the panel as the LD-decay was computed without correcting for population structure. Despite a lower number of markers on the D-genome, LD patterns follow a similar trend of declining LD-values over the genetic distances. Genome-wide and chromosome-wide LD decay is given in Fig. 3 and Supplementary Fig. S3, respectively.

### Genome-wide association analysis

**Figure 4:**
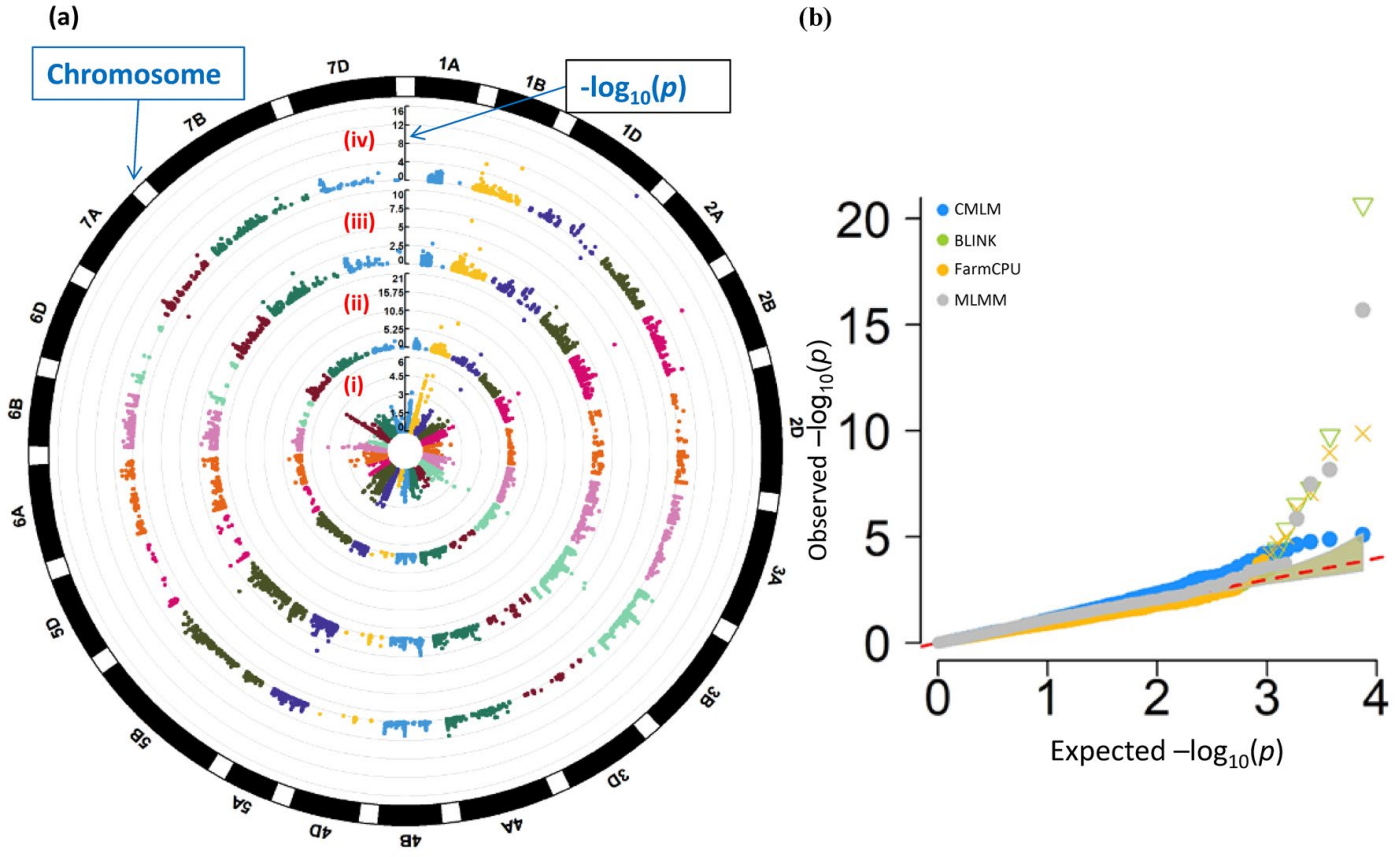
(**a**) Circular Manhattan plot displaying four methods of GWAS-analysis: (i) CMLM (ii) BLINK (iii) FarmCPU and (iv) MLMM. (**b**) QQ-plots of the observed and the expected *p* values of the GWAS models to visualize the false positives of the implemented models.

Four GWAS methods, CMLM, BLINK, FarmCPU and MLMM as implemented in GAPIT were used to identify marker-trait association (MTA). These four methods identified 25 significant MTAs with − log10 (*p* value) > 4.0 as shown in circular Manhattan plots (Table 1; Fig. 4a). All the MTAs identified using BLINK and MLMM methods had FDR-corrected *p* value  < 0.05. But in FarmCPU only six MTAs and four in CMLM method had FDR-corrected *p* value  < 0.05. Q-Q plots reflected that the distribution of observed association (*p* values) were close to the distribution of the expected associations (Fig. 4b). This means the methods implemented for GWAS were sufficiently stringent to control spurious associations.

**Table 1:** Details of significant MTA [− log10 (*p* value) > 4.0] obtained using different GWAS methods along with other details.

GWAS method	MTA	Chr (cM)^a^	Chr (bp)^b^	*P* value	MAF	FDR adjusted *P* values	Effect	Class^c^	Chr^c^
CMLM	AX-94470359*	1D (221)	1B (669,649,570)	7.88E−06	0.08041958	0.042173107	− 0.3847304	2	1B
	AX-95089267	1B (86)	1D (468,473,492)	1.31E−05	0.0979021	0.042173107	− 0.3817658	3	NA
	AX-94692978*	1B (24)	1D (38,789,852)	1.69E−05	0.4020979	0.042173107	0.1536129	2	1D
	AX-94478352	6B (36)	6B (26,625,397)	2.32E−05	0.08041958	0.043460881	− 0.4023998	2	6B
	AX-94809409*	3B (8)	3B (11,603,156)	3.98E−05	0.09440559	0.059631076	− 0.2455716	2	3B
	***AX-94635647***	1B (25)	1B (327,842,901)	5.10E−05	0.08391608	0.059669317	0.2517702	3	NA
	***AX-94554649***	1B (25)	1D (228,441,505)	5.66E−05	0.08741259	0.059669317	− 0.243364	2	1D
	***AX-95653365***	1B (25)	1A (296,212,973)	6.37E−05	0.09090909	0.059669317	0.2409785	NA	NA
BLINK	AX-94470359*	1D (221)	1B (669,649,570)	2.14E−21	0.08041958	1.60E−17	NA	2	1B
	AX-94723525*	1B (119)	1B (667,930,917)	1.68E−10	0.06643357	6.29E−07	NA	2	1B
	AX-94692978*	1B (24)	1D (38,789,852)	4.95E−08	0.4020979	0.00012353	NA	2	1D
	AX-94620141*	2B (27)	2D (14,772,766)	2.93E−07	0.48251748	0.0005495	NA	3	NA
	AX-94846537	1D (161)	1D (435,801,588)	4.23E−06	0.38111888	0.006336151	NA	3	NA
	AX-94809409*	3B (8)	3B (11,603,156)	3.58E−05	0.09440559	0.044692939	NA	2	3B
Farm CPU	AX-94470359*	1D (221)	1B (669,649,570)	1.35E−10	0.08041958	1.01E−06	− 0.3154022	2	1B
	AX-94412521	7A (30)	7D (379,062,494)	1.05E−09	0.08041958	3.94E−06	0.3087487	2	7D
	AX-94692978*	1B (24)	1D (38,789,852)	8.27E−08	0.4020979	0.00020648	0.1048849	2	1D
	AX-94620141*	2B (27)	2D (14,772,766)	4.90E−07	0.48251748	0.000916963	− 0.0983023	3	NA
	**AX-95232057**	3B (102)	3B (722,353,237)	9.23E−06	0.32167832	0.01382438	0.1091772	2	3B
	**AX-95630606**	3B (85)	3B (559,894,529)	2.03E−05	0.14335664	0.025397737	− 0.1335088	NA	NA
	AX-94920631	5A (43)	5A (9,652,511)	6.73E−05	0.30769231	0.072005474	0.0790124	2	5A
MLMM	AX-94470359*	1D (221)	1B (669,649,570)	2.04E−16	0.08041958	1.53E−12	NA	2	1B
	AX-94620141*	2B (27)	2D (14,772,766)	6.59E−09	0.48251748	2.47E−05	NA	3	NA
	AX-94723525*	1B (119)	1B (667,930,917)	3.23E−08	0.06643357	8.06E−05	NA	2	1B
	***AX-94640607***	1B (20)	1B (57,437,716)	1.36E−06	0.41258741	0.00254668	NA	2	1B

^a^Chromosome and genetic position in centimorgan as per^104^.

^b^Chromosome and physical position in base pairs as per IWGSC and 35K chip data information.

^c^Classification of SNP and potential chromosome on which this SNP is located as per^109^.

NA: Not available.

*MTA detected through more than one method.

Non-bold format represent novel MTA detected in the present study.

Bold format represent MTA underlying in QTL region for *P. thornei* and *P. neglectus* resistance as reported previously (shown in Supplementary Table S5).

Bold and italic format represents MTA detected near (~ 10.0 cM) to already identified marker regions for *P. thornei* and *P. neglectus* resistance through GWAS (shown in Supplementary Table S5).

CMLM analysis identified eight MTAs, with significant SNPs explaining approximately 23% to 26% of the total phenotypic variation. These MTAs were detected on chromosomes 1B, 1D, 3B and 6B. On chromosome 1B, five significant MTAs were identified and among these four were located between 24 and 25 cM (Table 1). Six MTAs were identified on chromosomes 1B, 1D, 2B and 3B using the BLINK method. Two MTAs were present on each of 1B and 1D whereas a single MTA was identified on 2B and 3B. Three of the MTAs located on chromosomes 1B (24 cM), 1D (221 cM) and 3B (8 cM) were common with those identified by CMLM (Table 1). FarmCPU analysis identified a total of seven MTAs on chromosomes 1B, 1D, 2B, 3B, 5A and 7A. Two MTAs identified on chromosome 3B and single MTA on each chromosome 1B, 1D, 2B, 5A and 7A. Two MTAs identified on chromosome 1D (221 cM) and 1B (24 cM) were also identified by both CMLM and BLINK methods (Table 1). MLMM analysis identified four MTAs on chromosomes 1B, 1D and 2B. Two MTAs detected on chromosome 1B and single MTA on each chromosome 1D and 2B. The MTA on chromosome 1D (221 cM) was identified in all the four methods as discussed above. MTA on 2B (27 cM) was detected in BLINK and FarmCPU methods whereas as MTA on 1B (119 cM) was detected only by BLINK method.

**Figure 5:**
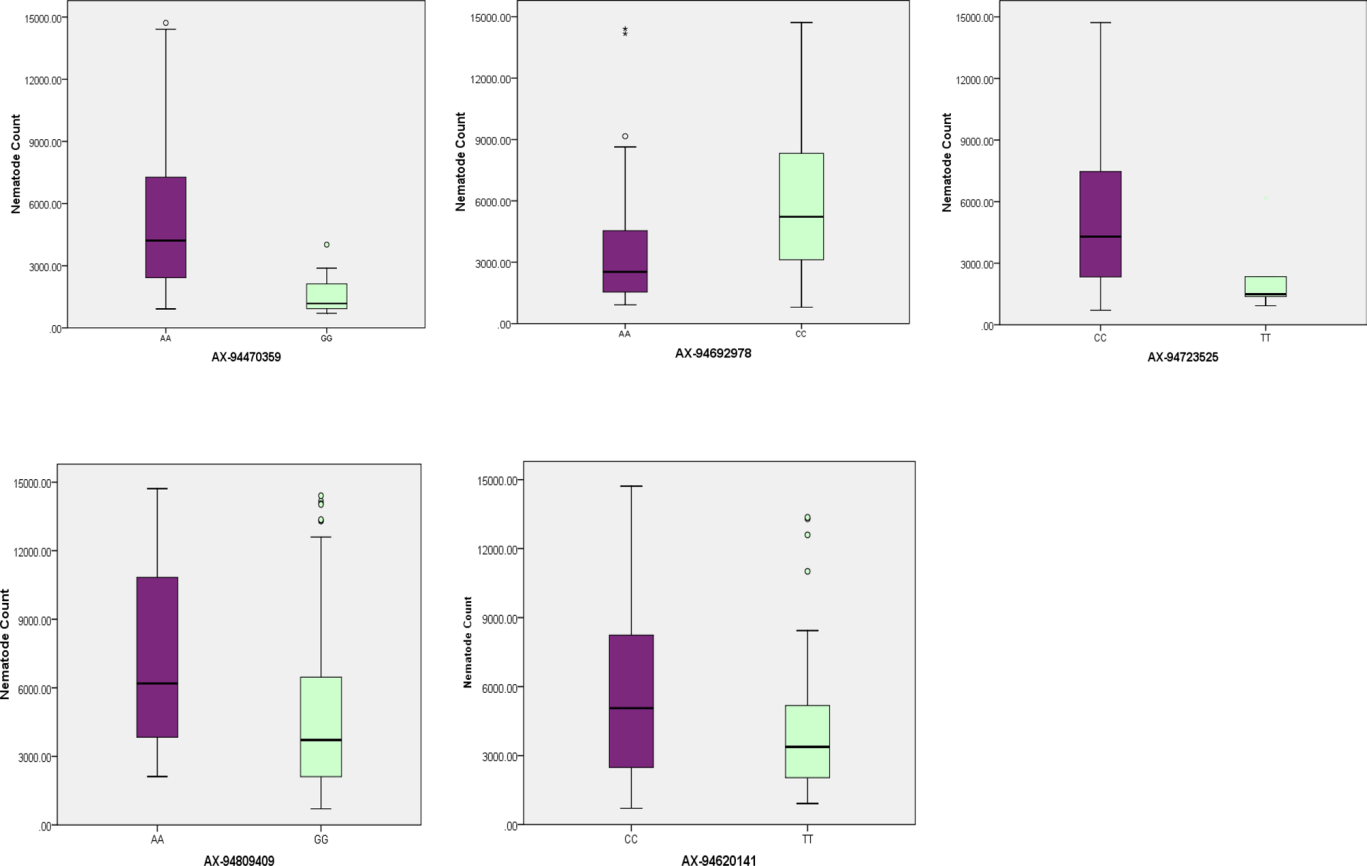
Boxplot showing the allelic effect of five significant MTAs for nematode count, X-axis representing the marker name and alleles, Y-axis depicts nematode count. Alleles which were decreasing or increasing the nematode count considered as favourable and unfavourable alleles, respectively.

Association analysis performed using all these four methods CMLM, BLINK, FarmCPU and MLMM seems to pick similar regions across the genome as discussed above. One of the MTAs (AX-94470359) on chromosome 1D located at 221 cM was detected in all four methods. MTA (AX-94692978) located on chromosome 1B at 24 cM was detected through CMLM, BLINK and FarmCPU. Likewise, MTA (AX-94620141) on chromosome 2B at 27 cM was detected through BLINK, FarmCPU and MLMM, respectively. These three MTAs on chromosomes 1D, 1B and 2B identified consistently in either three or four methods were considered to be the most significant of the study and could be useful for further analysis. Allelic effects of highly significant SNPs obtained consistently in more than one method is shown in the Fig. 5. Alleles leading to the decrease in nematode count were considered to be favourable alleles whereas unfavourable alleles increased the nematode count.

### Haplotype analysis

**Figure 6:**
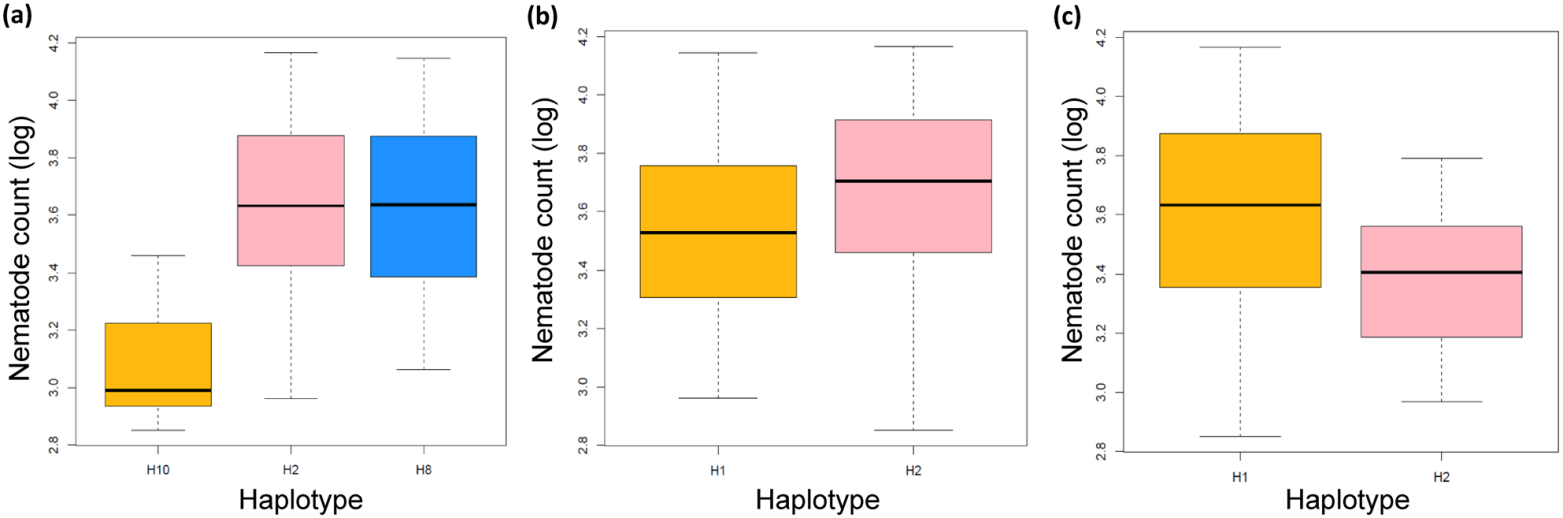
Association of the three LD blocks displaying the haplotypes nematode count as a box plots (**a**) LD block one with three haplotypes due to 9 significant SNPs (**b**) LD block two with two haplotypes due to 2 significant SNPs and (**c**) LD block three with two haplotypes due to 3 significant SNPs. Smaller haplotypes are not shown here.

We also generated the haplotypic blocks across the genome by using the SNPs. Interestingly, three main LD blocks that spanned around the regions of significant markers were used to generate haplotypes. Three large LD blocks and the corresponding haplotypes (LD block one, two and three) which were found to be associated with significant SNPs are given in Fig. 6.

LD block one was detected on the chromosome 1D at 28 cM (209–237 cM region). Three haplotypes were generated on the basis of genotyping results by using 132 genotypes (smaller haplotypes were not considered) (Fig. 6a). Haplotype 2 included 82 genotypes (3.63 ± 0.31), haplotype 8 included 41 genotypes (3.60 ± 0.30) and haplotype 10 included nine genotypes (3.09 ± 0.20). The mean value of nematode count was significantly lower (p < 0.01, t-test) in haplotype 10 (3.09 ± 0.20), than those of haplotype 8 (3.60 ± 0.30) and haplotype 2 (3.63 ± 0.31).

LD block two was also identified on chromosome 1D at 161 cM. Two haplotypes were generated using 140 genotypes (Fig. 6b). Haplotype 1 included 87 genotypes (3.54 ± 0.30) and haplotype 2 included 53 genotypes (3.66 ± 0.36). The mean value of nematode count was lower in haplotype 1 (3.54 ± 0.30) than the haplotype 2 (3.66 ± 0.36).

LD block three was detected on chromosome 1B at 1 cM (118–119 cM region). Two haplotypes were generated using 141 genotypes (Fig. 6c). Haplotype 1 included 127 genotypes (3.60 ± 0.33), haplotype 2 included 14 genotypes (3.39 ± 0.22). Haplotype 2 has a lower mean value of nematode count than haplotype 1 (3.60 ± 0.33).

### Genomic selection of the nematode counts

As nematode counting, used for resistance screening, is not straight forward and time consuming, we explored whether the genomic selection would predict the nematode count using genome-wide markers. For this analysis the population was divided into smaller subgroups starting from 20 to 80% of the population used as a training set and then genomic prediction was performed on the remaining set. We observed a prediction value of r = 0.40 to 0.44 when 56 to 70% of the population was used as a training set which is moderate for a complex trait like nematode count of the present study (Supplementary Fig. S4).

### Putative candidate gene associated with significant MTAs

The SNP tags for the highly significant MTAs were subjected to a BLAST search against recently released wheat genome sequence IWGSC RefSeq v1.0 (http://www.wheatgenome.org) to find the candidate genes. We identified 37 putative candidate genes (Supplementary Table S3), out of which nine genes having a significant role in disease resistance (including F-box-like domain superfamily, Leucine-rich repeat, cysteine-containing subtype, Cytochrome P450 superfamily, Zinc finger C2H2-type, RING/FYVE/PHD-type, etc.) (Table 2). No hit was found for the highly significant MTA (AX-94470359) located on chromosome 1D. SNP tags for markers AX-95630606 and AX-94478352 associated with 3B and 6B identified six putative candidate genes on each of the chromosomes. Similarly, more than one candidate genes were also identified for other MTAs. The maximum number of candidate genes 11 and 10 were detected on chromosome 1B and 3B, respectively (Supplementary Table S3).

**Table 2:** Details of disease related candidate gene showing putative associations with *P. thornei* resistance in wheat.

SNP Markers	SNP Tags	Gene ID	Chromosome	Start	End	Annotation
AX-94809409	TTAACTACTAGTGTTTCAAGCGTGCGATGCGACCA[A/G]GAAGAAGAAGACCCGTACTAGTTGACGATGTGAAC	TraesCS3B02G026600.1	3B	11,561,317	11,563,129	F-box associated interaction domain
AX-94846537	GGAGAGAAGGCCATGGTCGAGCTGAAGAAGTACTA[C/T]GCCATCTTCGATGAGAAGTGCCTGTCCAAGATTCC	TraesCS1D02G349500.1	1D	435,751,705	435,755,014	F-box domain
AX-94640607	CCAGATAGCTTTGCAAACCTGAGCACGAGTTGTTT[A/C]AGCTTCGGCATGGACCTCGAGATCGCTATTGCTTC	TraesCS1B02G073600.1	1B	57,437,259	57,439,274	F-box-like domain superfamily, Leucine-rich repeat, cysteine-containing subtype
AX-94620141	AGTGGACAACAGCCGTGTAGCCATGTTCCAGGATT[C/T]GCCGGTTTTGGCGGTAGAGCTCGACGTGGAAGAGA	TraesCS2B02G031700.1	2B	14,788,702	14,796,017	Cytochrome P450 superfamily
AX-95630606	GGGCCGAGTTTCAGTGTTCAGACTGGAGATCCATC[A/G]ATGGCCCGTTTGTGGGTGTGTGCGTCTACAACGTG	TraesCS3B02G349400.1	3B	559,877,229	559,878,277	ESCRT assembly domain, Vacuolar protein sorting-associated Vps28
AX-94723525	AGTCGAGTGACATCCATGGCTTCTCTGTCGATGAG[C/T]GCGGGGTGGACGCCGAAGCAGAACAAGCTGTTCGA	TraesCS1B02G449000.1	1B	667,917,663	667,918,731	Homeobox-like domain superfamily, SANT/Myb domain
		TraesCS1B02G449100.1	1B	667,930,685	667,931,664	Homeobox-like domain superfamily, SANT/Myb domain,
		TraesCS1B02G449200.1	1B	667,965,638	667,966,493	Homeobox-like domain superfamily, SANT/Myb domain
AX-94635647	TCATGATGCACATGAATATGAATCAGCCTTTAATC[C/T]CCAACGCATCATTCAGCTACCCTGTCCAACAAGAT	TraesCS1B02G181200.1	1B	327,842,228	327,845,323	Zinc finger C2H2-type, RING/FYVE/PHD-type

## Discussion

The use of resistant cultivar is considered as the economically feasible and eco-friendly method to control *P. thornei*^31^. Up to 69% and 32% reduction in wheat yield is reported due to *P. thornei* and *P. neglectus*, respectively^29,43^. Dababat et al.^31^ evaluated CIMMYT’s spring wheat genotypes for *P. thornei* resistance and identified 56 resistant lines under controlled environmental conditions in a pre-screen. These lines were further evaluated under natural field conditions and only 14 remained resistant. In the present study, 143 wheat genotypes (predominantly from India) were screened against *P. thornei* that showed a varied response when tested under controlled environmental conditions. No reference source of resistance in wheat against *P. thornei* is available in India. Kranti and Kanwar^19^ evaluated resistance and susceptibility in several wheat varieties (including Indian lines) for *P. thornei* resistance by the reproduction factor (Pf/Pi). The reproduction factor is commonly used in plant nematology as a quantitative value to assess resistance. In this study we found six resistant genotypes having reproduction factor (Pf/Pi) value equal or less than one, 24 moderately resistant genotypes (RF = 1–2), 25 moderately susceptible (RF = 2–3), 17 susceptible (RF = 3–4) and 71 highly susceptible (RF = more than 4) (Supplementary Table S4). In the current study, heritability (h^2^) was found to be 0.55, which showed moderate heritable value. This value indicates that despite the complexity of the trait which mainly owes to environmental conditions significant genetic effects were still observed and measured in the present study. Although in previous studies comparatively higher heritability for this nematode was also observed ranging from 0.83 to 0.97^36,44,45^. Dababat et al.^42^ detected very high heritability values, 0.95, 0.97 and 0.97 for *Heterodera avenae*, *P. neglectus* and *P. thornei*, respectively. Thus, in the future more experiments are needed to ascertain the phenotype and minimize the errors.

In the current study, our aim was to identify novel MTAs associated with *P. thornei* resistance by GWAS. With the rapid advancement in high throughput sequencing techniques, GWAS has been widely used to identify and dissect complex traits in wheat^46,47^. GWAS has been successfully used to study disease resistance in wheat^48–51^. GWAS was also performed in wheat for cereal cyst and root-lesion nematode resistance^31,42^. However, nothing from Indian wheat genotypes were screened and reported which links the phenotype to genotype. To avoid false association which is often present in the genotypes of local origin, effective corrections for population structure is needed^52–56^. Thus, we employed four methods of GWAS to locate the MTAs. In the present study, PCA detected two groups of the population indicating the existence of sub-populations. Interestingly we did not see major structuring of the populations due to the origin possibly suggesting despite a smaller group the genotypes were diverse.

Contrary to traditional linkage analysis, association mapping offers higher mapping resolution. In GWAS, diverse germplasm is used that contains a large number of recombination events as well as LD across a lineage that could help to detect mutations associated with phenotypic traits of interest^57^. The magnitude of LD is influenced by many factors like recombination rate, allele frequency, mating system, genetic isolation, population structure, selection, marker type^52,58–61^, etc. Many studies suggest that LD is not constant across the whole genome or along a single chromosome. It can occur over large distances but also decreases very quickly for nearby loci^62^. In our study, LD decay so observed was similar to earlier reports^63,64^ where LD decay in common wheat ranged between 0.22 to 10 cM using SNP markers^65^, 2 cM (in A and B genome) to 5 cM (in D genome) using SNP markers^61^. LD ranged from 8 cM (D genome) to 10 cM (A genome) and average LD for whole genome 5 cM was also reported^64^. The D-genome has a higher LD decay, which is believed to be mainly due to limited populations of *Aegilops tauschii* which contributed to the present wheat genome in the evolutionary history of wheat^66,67^.

For a complex trait like nematode resistance, using more than one GWAS analysis methods make results statistically more stringent and acceptable with higher confidence which could be further used for pre-breeding purposes. CMLM, FarmCPU, MLMM and BLINK were used to identify significant associations between genotypic and phenotypic data. In the present study, several common significant MTAs were detected through four association analysis methods. The mixed linear model has been widely used in controlling population structure and relatedness within GWAS and reduces the spurious associations^68–72^. However, mixed linear model-based methods are computationally challenging for large data sets. Hence, to overcome this problem, CMLM is implemented. CMLM method implemented in the GAPIT package decreases the effective sample size of such large and computationally challenging datasets by clustering individuals into groups^73,74^. CMLM improves statistical power by 5 to 15% and reduces computing time compared to regular MLM^73,75^. However, due to confounding between population structure, kinship and multiple testing corrections, CMLM based analysis can be insufficient, which could lead to a false association. Therefore, we also used FarmCPU analysis to mitigate this problem as the latter method has improved statistical power to detect causative genotype–phenotype associations and reduced false positives^76^. This method implements both the fixed-effect model (FEM) as covariates and a random effect model (REM) containing the kinship matrix. MLMM, which is a modified version of the mixed linear model, as it fits loci of large effect as covariates, detects more MTAs with smaller effects and has higher power and a lower FDR than single-locus approaches^77^. A recently developed BLINK method is also used in the present study to decrease the confounding problems that may lead to the spurious association^78^. BLINK implements a multiple loci test method by combining a fixed-effect model (FEM) with Bayesian information criteria (BIC). This method uses linkage disequilibrium (LD) information to replace the bin method.

Using four different GWAS methods, 25 MTAs were detected in association with *P. thornei* resistance. Among these, five MTAs on chromosome 1D (221 cM), 1B (24 cM), 3B (8 cM), 1B (119 cM) and 2B (27 cM) were obtained in more than one method. The SNP marker AX-94470359 on chromosome 1D detected in the present study was found common in all four methods. Among MTAs detected in the present study, seven were considered novel as detected for the first time whereas nine MTAs were found to be overlapped with or near to previously reported QTL/MTA regions (Table 1 and Supplementary Table S5).

Dababat et al.^42^ reported MTAs conferring resistance to *P. thornei* on chromosomes 1D. Similarly, Mulki et al.^79^ also identified QTL for *H. avenae* resistance on chromosome 1D in their genome-wide association study in wheat. In previous studies, several QTL for *P. thornei* resistance were also detected on chromosome 1B, 2B, and 3B^36–39,44,80,81^. In the CMLM method, SNP marker AX-95089267 was detected at position 86 cM on chromosome 1B. Previously a QTL, associated with susceptibility, was also identified between 60 and 67 cM on chromosome 1B region^80^. QTL/marker on chromosome 1B was also detected for another root-lesion nematode *P. neglectus* and cereal cyst nematode *H. avenae*^42,82,83^.

Another highly significant marker AX-94620141 was detected on chromosome 2B (27 cM) by MLMM, FarmCPU and BLINK methods. In a previous study by Zwart et al.^37^, a QTL for *P. thornei* resistance was detected on chromosome 2B at 27.9–35.1 cM interval. In a separate study by Linsell et al.^36^ three QTLs for *P. thornei* resistance were also identified on chromosome 2B, and mapped at 8.4–17.6 cM, 62.2–69.8 cM and 302.7- 306.8 cM, respectively. Recently, Rahman et al.^39^ performed fine mapping of *P. thornei* resistance loci on chromosome 2B of wheat. Although, MTA detected in our study is not on the same position as the fine mapped QTL, but its presence on the same chromosome suggests possible importance of chromosome 2B in resistance and thus can be considered as an important chromosome for nematode resistance. A candidate gene for Cytochrome P450 superfamily was also detected on this chromosome. Dababat et al.^42^ also detected the DArT marker for *P. neglectus* resistance on chromosome 2B.

We identified three markers [AX-94809409 (8 cM), AX-95232057 (102 cM), AX-95630606 (85 cM)] on chromosome 3B using CMLM, FarmCPU and BLINK methods. Schmidt et al.^80^ also identified QTL for *P. thornei* resistance in this region on chromosome 3B. Dababat et al.^42^ identified markers associated with *P. thornei* and *P. neglectus* resistance on chromosome 3B at 14.2 cM and 64.7 cM, respectively. Although, in previous studies, QTL/ markers associated with *H. avenae* and *P. neglectus* resistance were mapped on chromosome 5A, 6B and 7A^42,82,83^. In this study, two markers (AX-94920631 on chromosome 5A and AX-94478352 on chromosome 6B) were identified; no QTL was reported on these chromosomes for *P. thornei* resistance in previous studies.

In order to find out the allelic effect, the SNP alleles that decreased the nematode count were defined as favourable allele and those that resulted in an increase in nematode count were defined as unfavourable alleles. The allelic contribution may be quite effective in improving the resistance in wheat by marker-assisted breeding. The average phenotypic value (nematode count) of each significant SNP allele for nematode resistance was selected to calculate the allelic effect. Highly favorable allele detected in the present study can be used for the development of nematode resistant wheat genotypes.

Since the nematode count is a tedious trait that needs uprooting of the plants which is a cumbersome process specifically when dealing with large populations. That often is practically impossible when large populations are screened. Thus, we have conducted the genomic selection of the trait to investigate how well one could predict the phenotype of the nematode count without performing the phenotyping by using genomic-selection. We observed a prediction value of r = 0.40 to 0.44 when 56 to 70% of the population was used as a training set (Supplementary Fig. S4). These were not very high values but reasonable to use in the plant breeding practices when one targets multiple traits along with moderately heritable nematode resistance traits by employing relatively inexpensive genotyping instead of large costly phenotyping efforts. Small number of genotypes used in the current study further contributed to low-prediction values. However, despite using a small number of genotypes, we obtained a significant peak in the known region supported by old literature. Although, prediction values are low, information obtained from present study will provide a future roadmap to other studies that embark on genomic-selection using trait such as nematode count. Still, for future studies, we suggest inclusion of a higher number of genotypes to get better prediction values.

In the present study, 37 putative candidate genes were identified for significant MTAs (Supplementary Table S3). On the basis of the literature search, nine genes were detected having a putative role in disease resistance (Table 2). Important candidate genes identified (i) F-box domain; F-box protein plays a central role in plant immune response through hormone pathways such as salicylic acid and jasmonic acid pathway^84,85^. The plant uses these hormones in the regulation of defense response against several pests and pathogens^86^. Wheat F-box gene TafBA1 was also found to be involved in drought and heat tolerance^87,88^. (ii) Cytochrome P450 superfamily; Cytochrome P450s are heme-containing membrane-bound enzymes that involve in plant defense, and play an important role in disease response including fusarium head blight disease of wheat^89,90^, (iii) Vacuolar protein sorting-associated protein Vps 28 and Zinc finger protein; these are found to be drought-responsive genes and also play a role in other multiple stresses response in *Arabidopsis thaliana*^91^. (iv) Myb domain; Myb family is the largest family of a transcription factor in wheat. These are known to play a role in fungal infection caused by *Rhizoctonia cerealis* and also characterized as genes responsive to heat stress^92,93^.

## Conclusion

The results obtained in the present study should be highly useful for tapping resistance against *P. thornei* using natural genetic resources. Up to the best of our knowledge, only a single GWAS study is reported on *P. thornei* resistance in wheat^42^. The wheat genotypes, in the present study, showing resistance for *P. thornei* will be tested in the field under natural environmental conditions. For further confirmation, MTAs obtained shall be checked in appropriate segregating bi-parental population for potential breeding application in future. The SNP markers associated with significant MTAs can be used to develop KASP (Kompetitive Allele Specific PCR) markers, which will be further useful in marker-assisted breeding for *P. thornei* resistance. Further, we showed the effectiveness of the genomic-selection in successfully predicting the phenotype of the genotypes. Identified candidate genes need further validation through wet lab experiments.

## Materials and methods

### Plant material

The germplasm collection consisted of 143 wheat genotypes, predominantly collected from the pan-Indian wheat cultivation states, and was used for nematode screening and association analysis (Supplementary Table S6). Three resistant (Raj MR1, WH542, M6) and three susceptible (WH711, WH147, Opata) wheat genotypes were also included in the screening experiments as control.

### Nematode resistance test

Screening experiments were carried out using *P. thornei* population obtained from Indian Institute of Pulses Research (IIPR), Kanpur, India. Nematode population was maintained on carrot callus using the carrot disc method in the incubator at 22 °C for about three months^94^. Nematodes extraction was done from chopped carrot callus following modified Baermann’s funnel technique^95^. Seeds of wheat genotypes were surface sterilized with 0.2% (w/v) mercuric chloride (HgCl_2_) and then soaked in water for germination overnight. Germinated seeds were transferred to polyvinyl chloride (PVC) tubes (16 cm in height and 3.5 cm in diameter) filled with steam-sterilized soil. This soil was collected from the field, sieved and then sterilized twice at 121 °C for 2 h. At the bottom of each tube, a 20 μm sieve was fixed to prevent both root outgrowth and the nematode’s escape out of the tube. The tubes were placed in special holders on an irrigation system as described previously^96–99^. All the experiments were performed in the growth room under controlled environmental conditions (22 °C ± 2, 16 h light and 8 h darkness and ~ 65% relative humidity). Completely randomized design (CRD) with a minimum five replicates in five different batches (over two years) of each genotype was used. The plants were irrigated with Hoagland media at a fixed interval during the experiment.

Ten days post transplantation, each seedling was inoculated with 1000 *P. thornei* mixed stage juveniles and adult nematodes in solution by pipetting at a depth of 1.0 cm near to root. Plants were not watered for the next 3–4 days after inoculation of nematodes. Nematode extraction was done after 90 days of transplantation from soil and root by Cobb’s sieving and decanting method combined with modified Baermann’s funnel technique^95,100^. Nematode suspensions were collected and stored in bottles at 4 °C for counting. Three aliquots of 1.0 ml were taken from each bottle and nematodes were counted under a stereo microscope (Nikon SMZ645) at 40X-fold magnification. Reproduction factor (*P*f/*P*i) values (Pf means final population and Pi means an initial population of nematode) were calculated as the ratio between the number of nematodes counted at the end of the test and the number of nematodes used as inoculum on the basis of reproduction factor (Pf/Pi) values (Supplementary Table S4). Genotypes were categorized as resistant (Reproduction factor (RF) equal to or less than 1), moderately resistant (RF = 1–2), moderately susceptible (RF = 2–3), and susceptible (RF = 3–4) and highly susceptible (RF more than 4)^42^.

### Statistical analysis

In total, 2,860 data points (variety, batches and replication combinations) were generated. To control the errors all data points were manually checked and visually inspected by conducting outlier z-tests. The analysis of unbalanced nematode counts data was carried out by the mixed model using REML (restricted maximum likelihood)^101,102^. A REML model was later fitted using the ASREML-R library in R^103^. All terms are fitted as random and the best model was selected and fitted to predict the BLUPs of the varieties that were used in the subsequent GWAS analysis.

### Genotyping

From the seedling of the grown plants, leaf samples were collected for DNA extraction. All DNA samples were quantified on 0.8% agarose gel followed by quantification on a spectro-photometer-based Nanodropsystem. Affymetrix 35 K Axiom wheat Breeder chip was used for genotyping. We obtained 16,406 SNPs after quality control on this breeder chip. We further filtered the SNPs based on the minor allele frequency of 5%, heterozygosity (< 10%) and missing values that reduce the total SNP numbers to 7,491. Full set of 7,491 polymorphic SNPs that have genetic positions on the high-density consensus map was used for further analysis^104^.

### Population structure and Linkage disequilibrium analysis

To understand the population structure, PCA analysis was performed from a set of polymorphic SNPs implemented in TASSEL version 5.0 and displayed using R^103^. To compute pair-wise LD between the markers TASSEL version 5.2.54 was used. The pairwise LD was determined by using the full set of mapped 7,491 polymorphic and genetically anchored SNPs. To display whole genome and sub-genomic LD pairwise r^2^ versus distances in cM, sliding window of 50 SNPs was used and the graphics of the LD-decay were generated in R package GGPLOT2^105^.

### Genome-wide and haplotype-based association mapping

For the genome-wide association analysis of SNPs to nematode counts, GAPIT R package (3.0)^106^ was used. To compute the kinship matrix a set of thinned markers over the 21 wheat chromosomes were used to generate the IBS based kinship matrix. Four methods of GWAS analysis were used including compressed mixed linear model (CMLM)^73^, multi loci mixed linear model (MLMM)^77^, Fixed and random model Circulating Probability Unification (FarmCPU)^76^ and Bayesian-information and linkage-disequilibrium iteratively nested keyway (BLINK)^78^. In total, 7,491 SNPs obtained after filtering were used for genome-wide association analysis. To visualize the false positives of the implemented methods, QQ-plots were generated from each method in R (using the R-package R-CM plot). Manhattan plots were generated in R package qqman and CMplot. A robust threshold value of − log_10 _(*p* value) = 4.0 was set to reduce false positives and to ascertain the comparison across the implemented GWAS methods of this study. To compute the haplotype-based association analysis haplotypes were determined over the whole genome. PLINK version 1.9 was used to generate the LD-blocks which in turn is based on the default algorithms of finding haplotype blocks from Haploview software^107^.

### Genomic prediction analysis

A set of polymorphic, genetically mapped markers discussed above were used for the genomic selection. The analysis was conducted by a cross-validation approach wherein a set of training sets were drawn from the total population with a starting set of size 20 to 120 that corresponds to the 14 to 84% of the population. As there were only a few marker data sets missing which we imputed using mean values. A set of 500 cycles were run for this analysis as implemented in the software rrBLUP R-package^108^. The predicted regression coefficients obtained for these analyses were checked against the actual values and were reported in this study.

### Candidate gene analysis

To identify the potential candidate genes and their gene ontology, the sequences of SNPs defined significant association for *P. thornei* resistance, were searched for IWGSC sequence information in Ensembl plants for *T. aestivum* (http://plants.ensembl.org/Triticum_aestivum) and the high confidence genes were selected to explore the function. The protein products of genes present in the flanking sequence available for the SNP markers with maximum bases (50,000 bases before and after the SNP) were taken as putative genes.

## Supplementary Information

Supplementary Information.

Supplementary Tables.

## References

[1] Gupta, P. K., Balyan, H. S., Sharma, S. & Kumar, R. Genetics of yield, abiotic stress tolerance and biofortification in wheat (*Triticum aestivum* L.). *Theor. Appl. Genet.* **133**, 1569–1602 (2020). 10.1007/s00122-020-03583-3 32253477

[2] Yadav OP, Singh DV, Dhillon BS, Mohapatra T (2019). India’s evergreen revolution in cereals. Curr. Sci..

[3] Food and Agriculture Organization of the United Nations. Available online: http://www.fao.org/worldfoodsituation/csdb/en/ (accessed on 30 August 2020).

[4] Curtis T, Halford NG (2014). Food security: The challenge of increasing wheat yield and the importance of not compromising food safety. Ann. Appl. Biol..

[5] Nyaku, S. T., Affokpon, A., Danquah, A. & Brentu, F. C. Harnessing useful rhizosphere microorganisms for nematode control. In *Nematology–concepts, diagnosis and control* (eds. Shah, M. M. & Mahmood, M.) 153–182 (2017).

[6] Davis EL, Haegeman A, Kikuchi T, Jones J, Gheysen G, Fenoll C (2011). Degradation of the plant cell wall by nematodes. Genomics and Molecular Genetics of Plant–Nematode Interactions.

[7] Castillo P, Vovlas N (2007). Pratylenchus (Nematoda, Pratylenchidae): Diagnosis, biology, pathogenicity and management. Brill.

[8] Castillo P, Vovlas N, Jiménez-Díaz RM (1998). Pathogenicity and histopathology of *Pratylenchus thornei* populations on selected chickpea genotypes. Plant Pathol..

[9] Davis EL, MacGuidwin AE (2000). Lesion nematode disease. Plant Health Instr..

[10] Nicol JM, Rivoal R, Ciancio A, Mukerji KG (2008). Integrated management and biocontrol of vegetable and grain crops nematodes. Global Knowledge and Its Application for the Integrated Control and Management of Nematodes on Wheat.

[11] Nicol JM, Jones J, Gheysen G, Fenoll C (2011). Current nematode threats to world agriculture. Genomics and Molecular Genetics of Plant-Nematode Interactions.

[12] Owen KJ, Clewett TG, Bell KL, Thompson JP (2014). Wheat biomass and yield increased when populations of the root-lesion nematode (*Pratylenchus thornei*) were reduced through sequential rotation of partially resistant winter and summer crops. Crop Pasture Sci..

[13] Baghel KS, Singh R (2013). Alarming population of *Pratylenchus* spp. in chickpea growing areas in Rewa and its vicinity and its effect on plant growth and nodulation. Environ. Ecol..

[14] Ali SS, Sharma SB (2003). Nematode survey of chickpea production areas in Rajasthan, India. Nematol. Mediterr..

[15] Sebastian S, Gupta P (1995). Population dynamics of *Pratylenchus thornei* in infested fields at Allahabad. Indian J. Mycol. Plant Pathol..

[16] Tiwari SP, Vadhera I, Shukla BN, Bhatt J (1992). Studies on the pathogenicity and relative reactions of chickpea lines to Pratylenchus thornei (Filipjev, 1936) Sher and Allen, 1953. Indian J. Mycol. Plant Pathol..

[17] Dwivedi, K & Upadhyay, K. D. Nematode pests in rice-wheat-legume cropping systems in central Uttar Pradesh. In *Nematode pests in rice-wheat legume cropping systems*: Proceedings of a Regional Training Course, 1–5 September 1997, CCS Haryana Agricultural University, Hisar, Haryana, India (eds. Sharma S. B., Johansen C. & Midha S.K). Rice-Wheat Consortium Paper Series 4. New Delhi, India: Rice-Wheat Consortium for the Indo-Gangetic Plains 60–62 (1998).

[18] Ganguly AK, Pandey RN (2012). Severe damage caused by the root-lesion nematode, *Pratylenchus thornei*, in aerobic rice in India. Nematol. Mediterr..

[19] Kranti KVVS, Kanwar RS (2012). Evaluation of wheat varieties for resistance against *Pratylenchus thornei* and effect of sowing dates on its reproduction. Indian J. Nematol..

[20] Walia K, Kanwar RS, Bajaj HK (2005). Biodiversity of nemic fauna associated with wheat in two districts of Haryana. Indian J. Nematol..

[21] Hajihassani A, Davis RF, Timper P (2019). Evaluation of selected non fumigant nematicides on increasing inoculation densities of *Meloidogyne incognita* on cucumber. Plant Dis..

[22] Medina-Canales MG, Terroba-Escalante P, Manzanilla-López RH, Tovar-Soto A (2019). Assessment of three strategies for the management of *Meloidogyne arenaria *on carrot in Mexico using *Pochonia chlamydosporia* var. mexicana under greenhouse conditions. Biocontrol. Sci. Technol..

[23] Kim TY (2018). Nematicidal activity of grammicin produced by *Xylaria grammica* KCTC 13121BP against *Meloidogyne incognita*. Pest Manag. Sci..

[24] Xiang N, Lawrence KS, Donald PA (2018). Biological control potential of plant growth-promoting rhizobacteria suppression of *Meloidogyne incognita* on cotton and *Heterodera glycines* on soybean: A review. J. Phytopathol..

[25] Thompson JP, O’reilly MM, Clewett TG (2009). Resistance to the root-lesion nematode *Pratylenchus thornei* in wheat landraces and cultivars from the West Asia and North Africa (WANA) region. Crop Pasture Sci..

[26] Owen KJ, Clewett TG, Thompson JP (2010). Pre-cropping with canola decreased *Pratylenchus thornei* populations, arbuscular mycorrhizal fungi, and yield of wheat. Crop Pasture Sci..

[27] Cook R (1974). Nature and inheritance of nematode resistance in cereals. J. Nematol..

[28] Roberts PA, Starr JL, Cook R, Bridge J (2002). Concepts and consequences of resistance. Plant Resistance to Parasitic Nematodes.

[29] Thompson JP, Owen KJ, Stirling GR, Bell MJ (2008). Root-lesion nematodes (*Pratylenchus thornei* and *P. neglectus*): A review of recent progress in managing a significant pest of grain crops in northern Australia. Aust. Plant Pathol..

[30] Robinson NA, Sheedy JG, MacDonald BJ, Owen KJ, Thompson JP (2019). Tolerance of wheat cultivars to root-lesion nematode (*Pratylenchus thornei*) assessed by normalised difference vegetation index is predictive of grain yield. Ann. Appl. Biol..

[31] Dababat AA (2018). Host suitability of different wheat lines to *Pratylenchus thornei* under naturally infested field conditions in Turkey. Nematology.

[32] Thompson JP, Brennan PS, Clewett TG, Sheedy JG, Seymour NP (1999). Progress in breeding wheat for tolerance and resistance to root-lesion nematode (*Pratylenchus thornei*). Aust. Plant Pathol..

[33] Ogbonnaya FC (2008). Mining synthetic hexaploids for multiple disease resistance to improve bread wheat. Aust. J. Agric. Res..

[34] Sheedy JG, Thompson JP, Kelly A (2012). Diploid and tetraploid progenitors of wheat are valuable sources of resistance to the root lesion nematode *Pratylenchus thornei*. Euphytica.

[35] Sheedy JG, Thompson JP (2009). Resistance to the root-lesion nematode *Pratylenchus thornei* of Iranian landrace wheat. Aust. Plant Pathol..

[36] Linsell KJ (2014). QTL for resistance to root lesion nematode (*Pratylenchus thornei*) from a synthetic hexaploid wheat source. Theor. Appl. Genet..

[37] Zwart RS (2010). QTL mapping of multiple foliar disease and root-lesion nematode resistances in wheat. Mol. Breed..

[38] Toktay H, McIntyre CL, Nicol JM, Ozkan H, Elekcioglu HI (2006). Identification of common root-lesion nematode (*Pratylenchus thornei* Sher et Allen) loci in bread wheat. Genome.

[39] Rahman MS (2019). Fine mapping of root lesion nematode (*Pratylenchus thornei)* resistance loci on chromosomes 6D and 2B of wheat. Theor. Appl. Genet..

[40] Williams KJ (2002). Mapping of the root lesion nematode (*Pratylenchus neglectus*) resistance gene *Rlnn1* in wheat. Theor. Appl. Genet..

[41] Würschum T (2012). Comparison of biometrical models for joint linkage association mapping. Heredity.

[42] Dababat AA (2016). Association analysis of resistance to cereal cyst nematodes (*Heterodera avenae*) and root lesion nematodes (*Pratylenchus neglectus* and *P. thornei*) in CIMMYT advanced spring wheat lines for semi-arid conditions. Breed. Sci..

[43] McDonald AH, Nicol JM, Luc M, Sikora RA, Bridge J (2005). Nematode parasites of cereals. Plant Parasitic Nematodes in Subtropical and Tropical Agriculture.

[44] Zwart RS, Thompson JP, Sheedy JG, Nelson JC (2006). Mapping quantitative trait loci for resistance to *Pratylenchus thornei* from synthetic hexaploid wheat in the International Triticeae Mapping Initiative (ITMI) population. Aust. J. Agric. Res..

[45] Thompson JP, Zwart RS, Butler D (2012). Inheritance of resistance to root-lesion nematodes (*Pratylenchus thornei* and *P. neglectus*) in five doubled-haploid populations of wheat. Euphytica.

[46] Sun CW (2017). Genome-wide association study for 13 agronomic traits reveals distribution of superior alleles in bread wheat from the yellow and Huai Valley of China. Plant Biotechnol. J..

[47] Oyiga BC (2018). Allelic variations and differential expressions detected at quantitative trait loci for salt stress tolerance in wheat. Plant Cell Environ..

[48] Adhikari TB, Jackson EW, Gurung S, Hansen JM, Bonman JM (2011). Association mapping of quantitative resistance to *Phaeosphaeria nodorum* in spring wheat landraces from the USDA National Small Grains Collection. Phytopathology.

[49] Hao CY (2012). Association mapping and haplotype analysis of a 3.1-Mb genomic region involved in Fusarium head blight resistance on wheat chromosome 3BS. PLoS ONE.

[50] Kollers S (2013). Whole genome association mapping of Fusarium head blight resistance in European winter wheat (*Triticum aestivum* L.). PLoS ONE.

[51] Letta T (2013). Searching for novel sources of field resistance to Ug99 and Ethiopian stem rust races in durum wheat via association mapping. Theor. Appl. Genet..

[52] Flint-Garcia SA, Thornsberry JM, Buckler ES (2003). Structure of linkage disequilibrium in plants. Annu. Rev. Plant Biol..

[53] Yu J (2006). A unified mixed-model method for association mapping that accounts for multiple levels of relatedness. Nat. Genet..

[54] Kang HM (2008). Efficient control of population structure in model organism association mapping. Genetics.

[55] Stich B (2008). Comparison of mixed-model approaches for association mapping. Genetics.

[56] Larsson SJ, Lipka AE, Buckler ES (2013). Lessons from dwarf8 on the strengths and weaknesses of structured association mapping. PLoS Genet..

[57] Burghardt LT, Young ND, Tiffin P (2017). A guide to genome-wide association mapping in plants. Curr. Protoc. Plant. Biol..

[58] Ardlie KG, Kruglyak L, Seielstad M (2002). Patterns of linkage disequilibrium in the human genome. Nat. Rev. Genet..

[59] Wray NR, Purcell SM, Visscher PM (2011). Synthetic associations created by rare variants do not explain most GWAS results. PLoS Biol..

[60] Yang J, Shikano T, Li MH, Merilä J (2014). Genome-wide linkage disequilibrium in nine-spined stickleback populations. G3.

[61] Sukumaran S, Dreisigacker S, Lopes M, Chavez P, Reynolds MP (2015). Genome-wide association study for grain yield and related traits in an elite spring wheat population grown in temperate irrigated environments. Theor. Appl. Genet..

[62] Neumann K, Kobiljski B, Denčić SS, Varshney RK, Börner A (2011). Genome-wide association mapping: A case study in bread wheat (*Triticum aestivum* L.). Mol. Breed..

[63] Hu W (2020). Genome-wide association mapping revealed syntenic loci QFhb-4AL and QFhb-5DL for Fusarium head blight resistance in common wheat (*Triticum aestivum* L.). BMC Plant Biol..

[64] Sehgal D (2017). Identification of genomic regions for grain yield and yield stability and their epistatic interactions. Sci. Rep..

[65] Zegeye H, Rasheed A, Makdis F, Badebo A, Ogbonnaya FC (2014). Genome-wide association mapping for seedling and adult plant resistance to stripe rust in synthetic hexaploid wheat. PLoS ONE.

[66] Edae EA, Byrne PF, Haley SD, Lopes MS, Reynolds MP (2014). Genome-wide association mapping of yield and yield components of spring wheat under contrasting moisture regimes. Theor. Appl. Genet..

[67] Kahl SM, Ulrich A, Kirichenko AA, Müller MEH (2015). Phenotypic and phylogenetic segregation of *Alternaria infectoria* from small-spored Alternaria species isolated from wheat in Germany and Russia. J. Appl. Microbiol.

[68] Wallace JG, Larsson SJ, Buckler ES (2014). Entering the second century of maize quantitative genetics. Heredity.

[69] Lipka AE (2013). Genome-wide association study and pathway-level analysis of tocochromanol levels in maize grain. G3.

[70] Wei L (2016). Genome-wide association analysis and differential expression analysis of resistance to Sclerotinia stem rot in *Brassica napus*. Plant Biotechnol. J..

[71] Chang HX, Brown PJ, Lipka AE, Domier LL, Hartman GL (2016). Genome-wide association and genomic prediction identifies associated loci and predicts the sensitivity of tobacco ringspot virus in soybean plant introductions. BMC Genom..

[72] Crowell S (2016). Genome-wide association and high-resolution phenotyping link *Oryza sativa* panicle traits to numerous trait-specific QTL clusters. Nat. Commun..

[73] Zhang Z (2010). Mixed linear model approach adapted for genome-wide association studies. Nat. Genet..

[74] Lipka AE (2012). GAPIT: Genome association and prediction integrated tool. Bioinformatics.

[75] Tang Y (2016). GAPIT version 2: An enhanced integrated tool for genomic association and prediction. Plant Genome.

[76] Liu X, Huang M, Fan B, Buckler ES, Zhang Z (2016). Iterative usage of fixed and random effect models for powerful and efficient genome-wide association studies. PLoS Genet..

[77] Segura V (2012). An efficient multi-locus mixed-model approach for genome-wide association studies in structured populations. Nat. Genet..

[78] Huang M, Liu X, Zhou Y, Summers RM, Zhang Z (2018). BLINK: A package for the next level of genome-wide association studies with both individuals and markers in the millions. GigaScience.

[79] Mulki, M., A. *et al.* Association mapping for soil borne pathogen resistance in synthetic hexaploid wheat. *Mol. Breed*. **31,** 299–311 (2013).

[80] Schmidt AL, McIntyre CL, Thompson J, Seymour NP, Liu CJ (2005). Quantitative trait loci for root lesion nematode (*Pratylenchus thornei*) resistance in Middle-Eastern landraces and their potential for introgression into Australian bread wheat. Aust. J. Agric. Res..

[81] Zwart RS, Thompson JP, Godwin ID (2005). Identification of quantitative trait loci for resistance to two species of root-lesion nematode (*Pratylenchus thornei* and *P. neglectus*) in wheat. Aust. J. Agric. Res..

[82] Williams KJ, Willsmore KL, Olson S, Matic M, Kuchel H (2006). Mapping of a novel QTL for resistance to cereal cyst nematode in wheat. Theor. Appl. Genet..

[83] Jayatilake DV (2015). Genetic mapping of the *Cre8* locus for resistance against cereal cyst nematode (*Heterodera avenae* Woll.) in wheatGenetic mapping of the *Cre8* locus for resistance against cereal cyst nematode (*Heterodera avenae* Woll.) in wheat. Mol. Breed..

[84] Yu H, Wu J, Xu N, Peng M (2007). Roles of F-box proteins in plant hormone responses. Acta. Biochim. Biophys. Sin..

[85] Odilbekov F, Armoniené R, Koc A, Svensson J, Chawade A (2019). GWAS-assisted genomic prediction to predict resistance to septoria tritici blotch in nordic winter wheat at seedling stage. Front. Genet..

[86] Bari R, Jones JD (2009). Role of plant hormones in plant defence responses. Plant Mol. Biol..

[87] Kong X (2016). Stress-inducible expression of an F-box gene TaFBA1 from wheat enhanced the drought tolerance in transgenic tobacco plants without impacting growth and development. Front. Plant Sci..

[88] Li Q (2018). Wheat F-box protein gene TaFBA1 is involved in plant tolerance to heat stress. Front. Plant Sci..

[89] Walter S (2008). Components of the gene network associated with genotype-dependent response of wheat to the Fusarium mycotoxin deoxynivalenol. Funct. Integr. Genomic..

[90] Walter S, Doohan F (2011). Transcript profiling of the phytotoxic response of wheat to the Fusarium mycotoxin deoxynivalenol. Mycotoxin Res..

[91] Huang D, Wu W, Abrams SR, Cutler AJ (2008). The relationship of drought-related gene expression in *Arabidopsis thaliana* to hormonal and environmental factors. J. Exp. Bot..

[92] Zhao Y (2017). Characterization of wheat MYB genes responsive to high temperatures. BMC Plant Biol..

[93] Shan T (2016). The wheat R2R3-MYB transcription factor TaRIM1 participates in resistance response against the pathogen *Rhizoctonia cerealis* infection through regulating defense genes. Sci. Rep..

[94] Moody EH, Lownsbery BF, Ahmed JM (1973). Culture of the root-lesion nematode *Pratylenchus vulnus* on carrot disks. J. Nematol..

[95] Schindler A (1961). A simple substitute for a Baermann funnel. Plant Dis. Rep..

[96] Keil T, Laubach E, Sharma S, Jung C (2009). Screening for resistance in the primary and secondary gene pool of barley against the root-lesion nematode *Pratylenchus neglectus*. Plant Breed..

[97] Sharma S (2011). QTL analysis of root-lesion nematode resistance in barley: 1. *Pratylenchus neglectus*. Theor. Appl. Genet..

[98] Sharma S, Sharma S, Keil T, Laubach E, Jung C (2011). Screening of barley germplasm for resistance to root lesion nematodes. Plant. Gen. Res..

[99] Galal A (2014). Comparative QTL analysis of root lesion nematode resistance in barley. Theor. Appl. Genet..

[100] Cobb, N. A. Estimating the nema population of the soil. USDA Agricultural Technology Circular I. Bureau of Plant Industry, Office of Technology. *US Department of Agriculture (*1918).

[101] Patterson HD, Thompson R (1971). Recovery of inter-block information when block sizes are unequal. Biometrika.

[102] Volpato L (2019). Multi-trait multi-environment models in the genetic selection of segregating soybean progeny. PLoS ONE.

[103] R Core Team. R: A language and environment for statistical computing. R Foundation for Statistical Computing, Vienna. http://www.R-project.org/ (2013).

[104] Allen AM (2017). Characterization of a Wheat Breeders’ Array suitable for high-throughput SNP genotyping of global accessions of hexaploid bread wheat (*Triticum aestivum*). Plant Biotechnol. J..

[105] Wickham H (2016). ggplot2: Elegant Graphics for Data Analysis.

[106] Wang, J. & Zhang, Z. GAPIT version 3: An interactive analytical tool for genomic association and prediction. *Preprint* (2018).

[107] Purcell S (2007). PLINK: A tool set for whole-genome association and population-based linkage analyses. Am. J. Hum. Genet..

[108] Endelman JB (2011). Ridge regression and other kernels for genomic selection with R package rrBLUP. Plant Genome.

[109] Lange TM, Heinrich F, Enders M, Wolf M, Schmitt AO (2020). In silico quality assessment of SNPs—A case study on the Axiom^®^ Wheat genotyping arrays. Curr. Plant Biol..

