# Supplementary material for: Genome-wide association study in hexaploid wheat identifies novel genomic regions associated with resistance to root lesion nematode (Pratylenchus thornei)
Source: Sci Rep. 2021 Feb 11;11:3572. doi: 10.1038/s41598-021-80996-0 (PMC7878755; doi:10.1038/s41598-021-80996-0)
Supplement: Supplementary file 1 — Supplementary Information. [file 41598_2021_80996_MOESM1_ESM.pdf]

**Supplementary Figures**

# Genome-Wide Association Study in Hexaploid Wheat Identifies Novel Genomic Regions Associated with Resistance to Root Lesion Nematode (*Pratylenchus thornei*)

Deepak Kumar[1,2], Shiveta Sharma[1], Rajiv Sharma[3], Saksham Pundir[1,2], Vikas Kumar Singh[1], Deepti Chaturvedi[1], Bansa Singh[4], Sundeep Kumar[5], Shailendra Sharma[1]*

1. Department of Genetics and Plant Breeding, Chaudhary Charan Singh University (CCSU), Meerut 250 004, Uttar Pradesh, India.
2. Department of Botany, Chaudhary Charan Singh University (CCSU), Meerut 250 004, Uttar Pradesh, India.
3. Scotland's Rural College (SRUC), Peter Wilson Building, West Mains Road, Edinburgh EH9 3JG United Kingdom
4. Division of Crop Protection, Indian Institute of Pulses Research (IIPR), Kanpur - 208 024, Uttar Pradesh, India
5. Division of Genomic Resources, National Bureau of Plant Genetic Resources (NBPGR), Pusa campus, New Delhi-110 012, India

*Corresponding author:
Shailendra Sharma
Department of Genetics and Plant Breeding,
Chaudhary Charan Singh University,
Meerut-250004 (U.P), India
Email: Shgjus6@gmail.com

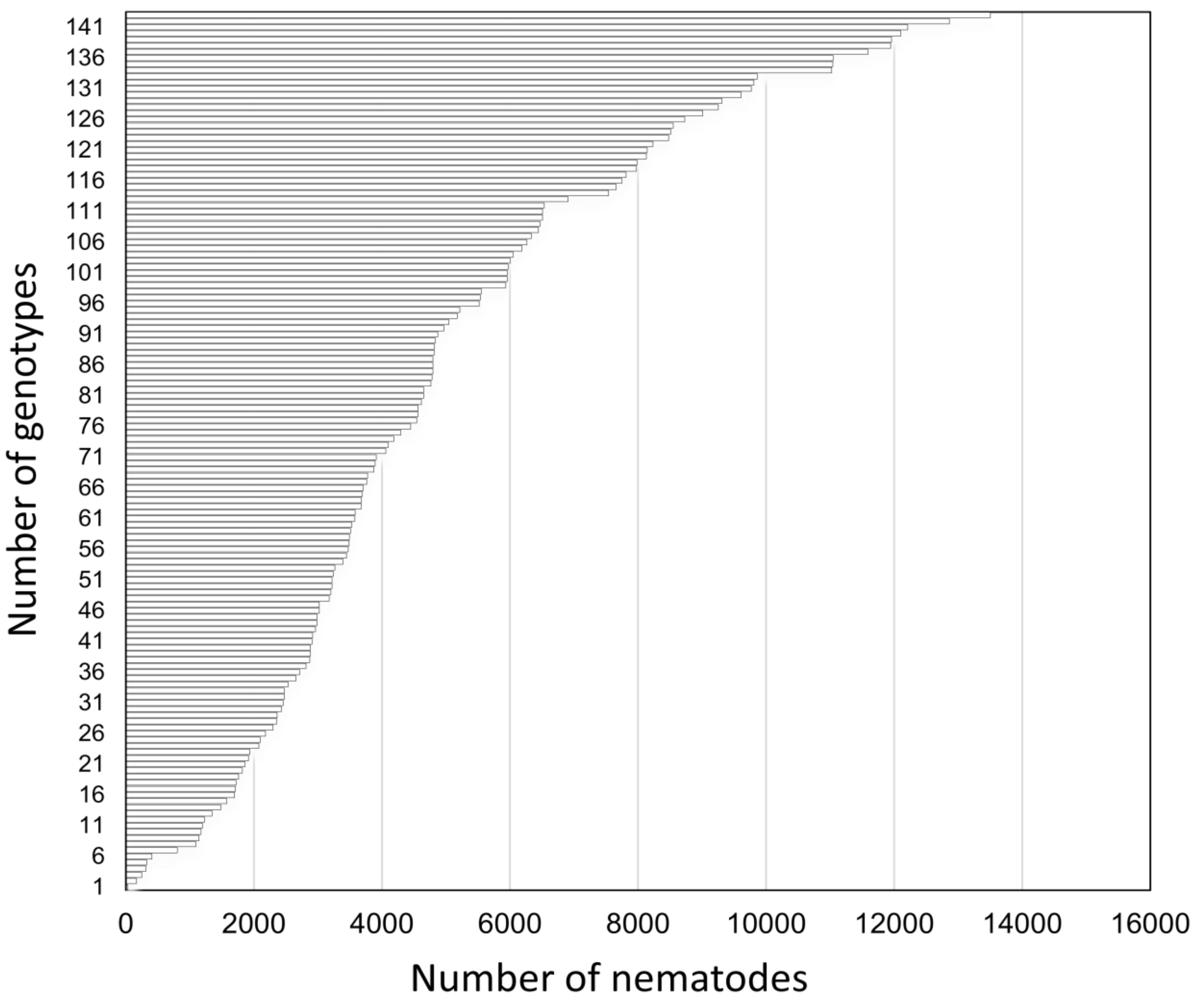


**Figure S1.** Histogram depicting the distribution of nematodes among 143 wheat genotypes.

.

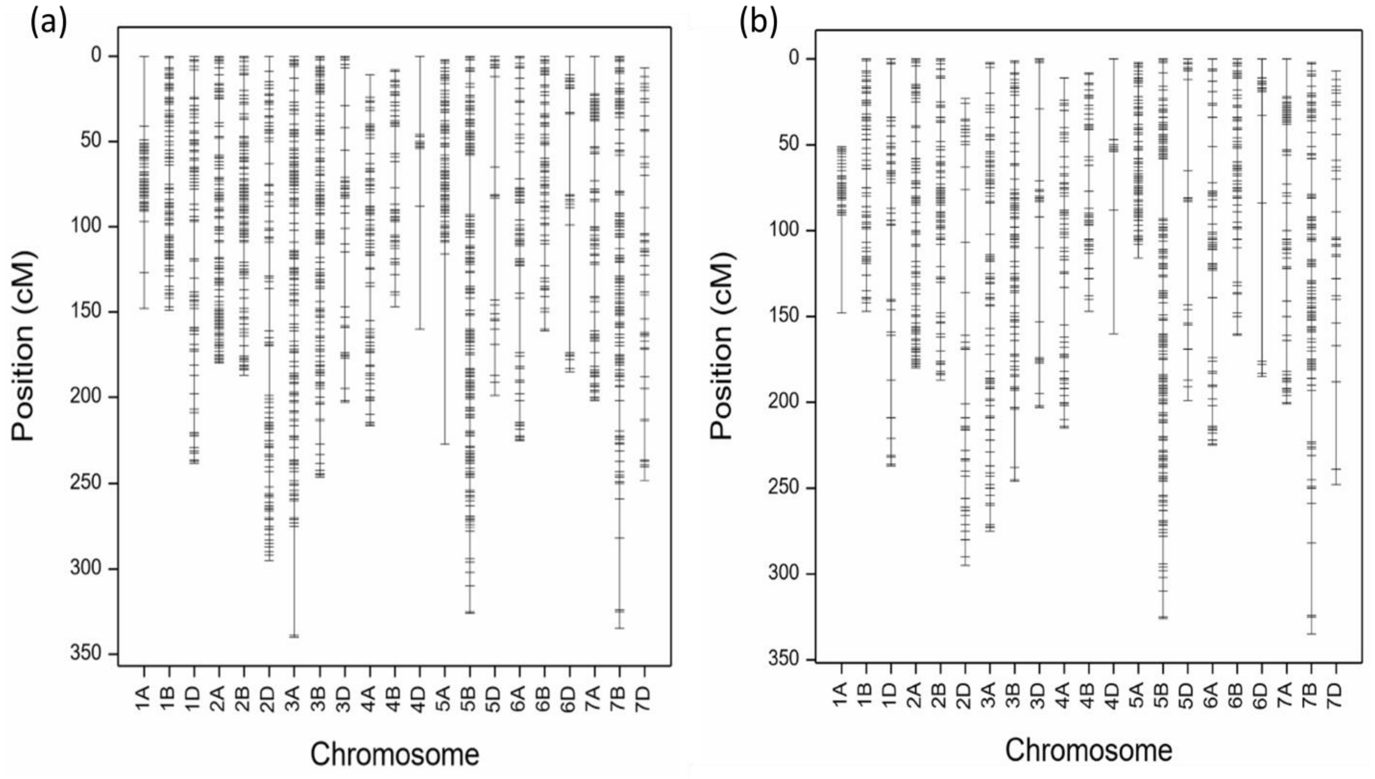


**Figure S2.** Distribution of SNPs on wheat chromosomes before filtration (a) and after filtration (b).

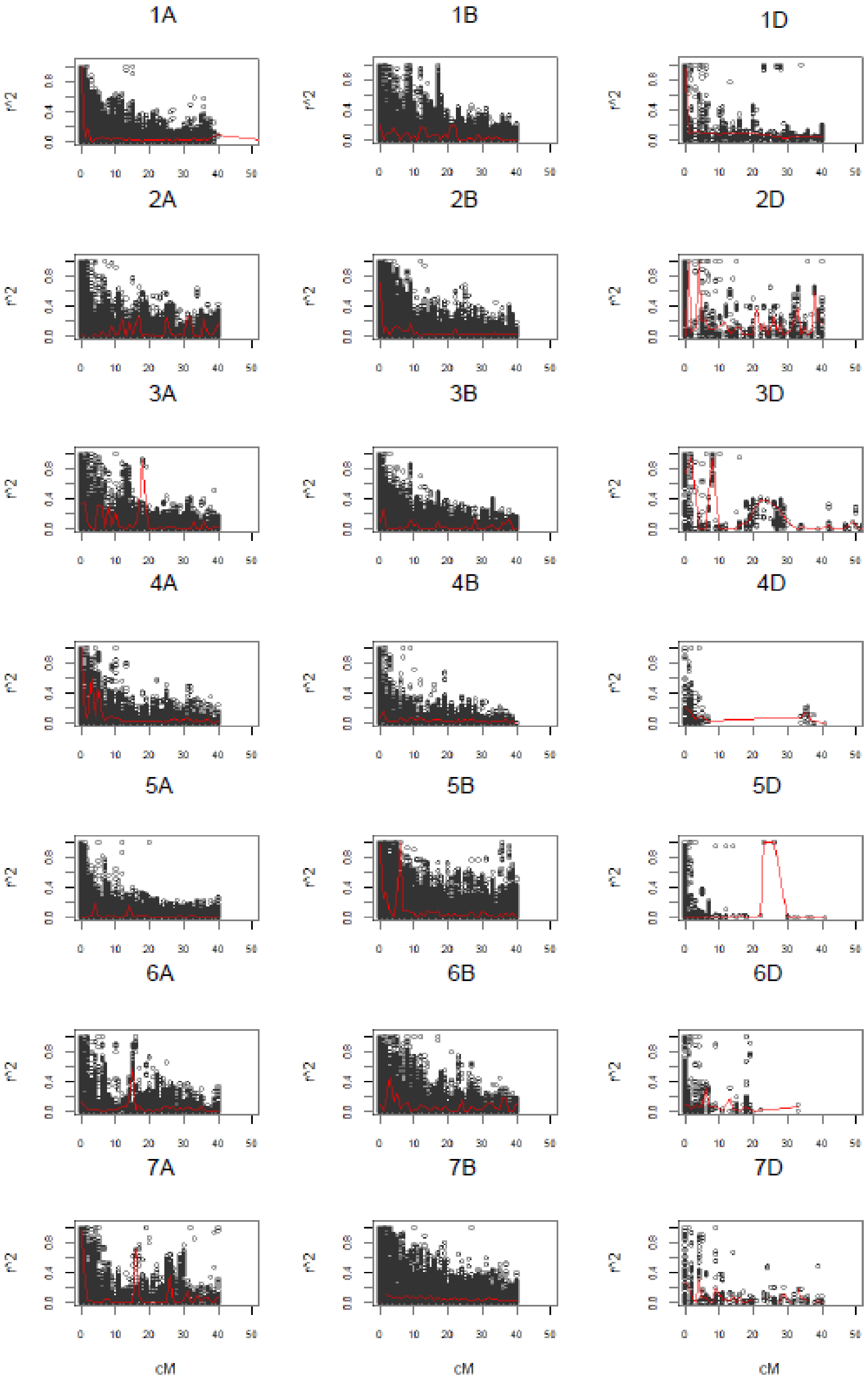


**Figure S3.** Chromosome wide LD deacy measured as r-squared against genetic distance between pairs of SNPs. LOESS curve (red color) was fitted showing that LD decays with genetic map distance.

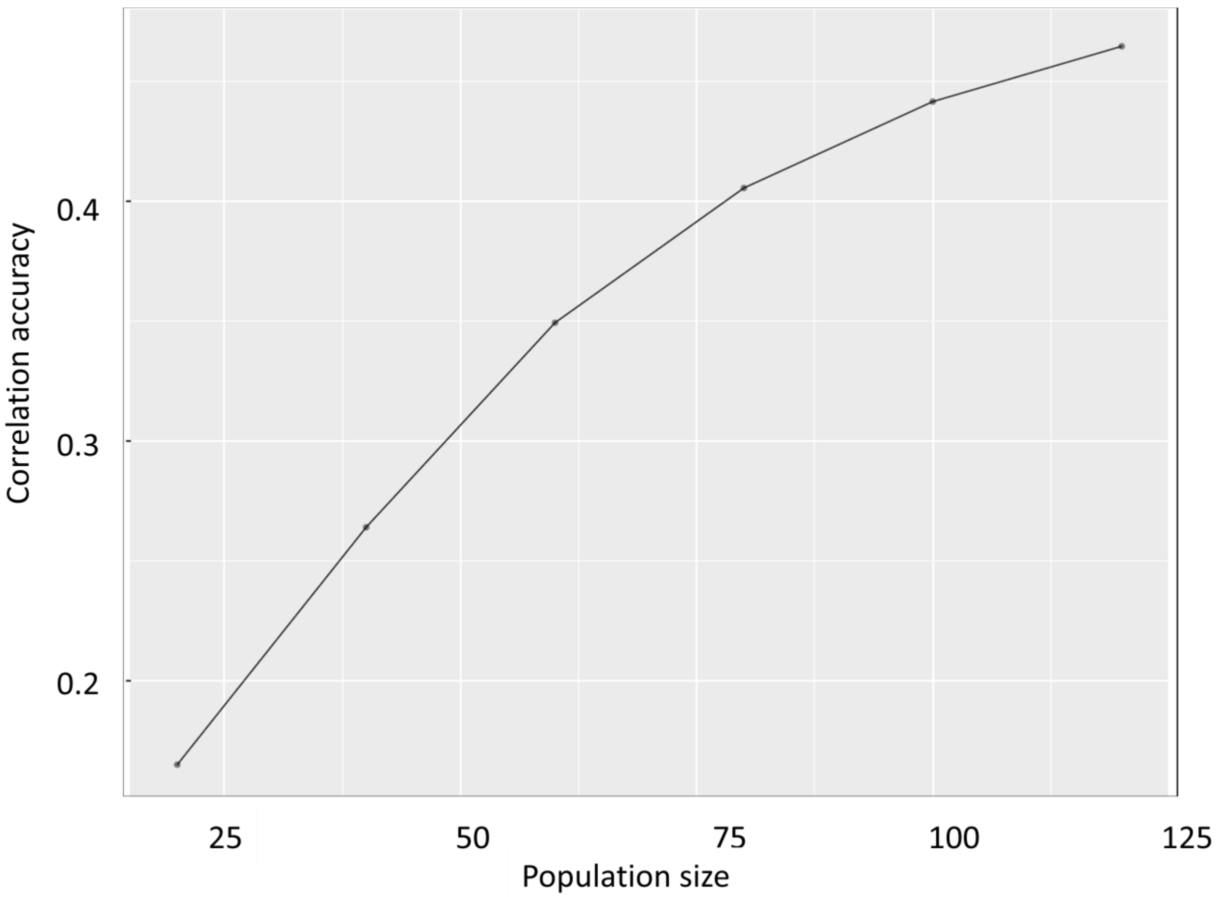


**Figure S4.** Genomic prediction of the nematode count and accuracy using set of polymorphic genetically mapped markers.
